# Comparison of Weight Reduction, Change in Parameters and Safety of a Very Low Carbohydrate Diet in Comparison to a Low Carbohydrate Diet in Obese Japanese Subjects with Metabolic Disorders

**DOI:** 10.3390/nu15061342

**Published:** 2023-03-09

**Authors:** Takako Kikuchi, Akifumi Kushiyama, Miho Yanai, Chieko Kashiwado, Takeshi Seto, Masato Kasuga

**Affiliations:** 1 The Institute of Medical Science, Asahi Life Foundation, Tokyo 103-0002, Japan; 2 Department of Pharmacotherapy, Meiji Pharmaceutical University, Kiyose 204-8588, Japan; 3 RIZAP GROUP, Inc., Tokyo 160-0023, Japan

**Keywords:** diet, LCD, VLCD, randomized study, metabolic disorders

## Abstract

Recently, low-carbohydrate diets (LCDs) have gained worldwide attention. LCDs are potentially effective for Japanese overweight and obese individuals with metabolic disorders. However, few randomized trials of LCDs have focused on the difference between LCDs and VLCDs. We conducted a randomized, prospective study of 42 Japanese, obese adults aged 28–65 years to evaluate the efficacy and safety of LCD and VLCD. To ensure the accuracy of the study, all test meals were provided, and compliance was checked using a smartphone app. Body composition measurements and blood tests were performed before and after the 2-month dietary intervention. The results showed that both methods significantly reduced body weight and fat, and also improved lipid abnormalities and liver function. In the current study, the reductions in weight and fat were comparable. The results of a questionnaire at the end of the study indicated that the LCD was easier to carry out than the VLCD, suggesting that the LCD was sustainable. The present study was unique in that it was a randomized, prospective study of Japanese subjects and that accurate data were obtained by providing meals.

## 1. Introduction

Obesity, a global problem, is associated with disease, including adult diseases in productive middle-aged and older adults [1]. In Japan, according to the statistics of the Ministry of Health, Labor, and Welfare (2019), 33% of Japanese men had a BMI of ≥25 kg/m^2^. Obesity is particularly prevalent in the 40- to 60-year age group. In addition, Japanese people are susceptible to the development of type 2 diabetes and metabolic diseases when they gain weight and accumulate visceral fat, partly due to their genetic background [2]. Therefore, the definition of obesity in Japan is ≥BMI 25 kg/m^2^, which is lower in comparison to Western countries. In our previous report, BMI in patients with type 2 diabetes has been increasing for several decades, and obesity is getting problematic in Japan [3]. 

Nutrition therapy is indispensable for proper weight management. Recently, low-carbohydrate diets (LCDs) have gained worldwide attention. Specifically, the American Diabetes Association (ADA) consensus report [4] lists multiple choices for diet therapies, including LCDs. In Japan, the Japan Diabetes Society previously recommended a calorie-restricted diet as the only dietary option for diabetes therapy; the latest guideline issued in 2019 stated that an LCD is not recommended because there is little evidence of its efficacy for Japanese subjects. 

In an LCD, the amount of carbohydrates ranges from 20 g to 130 g. A review published in 2008 [5] defined two LCDs: a very low carbohydrate diet (VLCD; 20–50 g/day) and an LCD (<130 g/day). In a previous self-control trial in Japan, an LCD was shown to be effective for lowering body weight (BW), HbA1c and triglyceride levels in patients with type 2 diabetes [6,7]. LCDs are potentially effective for Japanese overweight and obese patients with type 2 diabetes. However, few randomized trials of LCDs have focused on the difference between LCDs and VLCDs. In addition, many diet studies [8] rely on self-reporting over a 3- to 7-day period to assess dietary intake. As a result, the quantification of the amount of food intake throughout the trial period is probably inaccurate, and the appropriate amount of carbohydrate intake in an LCD or VLCD is still unknown. 

We hypothesized that a VLCD would be more effective than an LCD in treating obesity in Japanese overweight or obese individuals with metabolic disorders. In this study, to ensure accuracy, subjects were randomly assigned to receive a diet with carbohydrate intake restricted to 120 g/day (LCD) or 50 g/day (VLCD). We provided test meals for all subjects throughout the study period and used a real-time smart phone app (RIZAP ONLINE^®^) to monitor each subject’s diet, which was developed and adopted as an original assessment method. 

## 2. Materials and Methods

This study was a single-center, 2-month, comparative, two-arm, randomized, open-label trial performed between 12 July 2017, and 10 November 2017. The protocol was approved by the Committee of Ethics in the Institute of Medical Science, Asahi Life Foundation (approval number 08902). Informed consent was obtained from all subjects involved in the study. Investigations were carried out in accordance with the principals of the Declaration of Helsinki. The subjects consisted of 42, obese (median BMI 28.4 kg/m^2^), Japanese men and women of 28–65 years of age who were recruited through two local businesses in Tokyo. The inclusion criteria were as follows: (1) age 20–65 years; (2) BMI 25 to <35 kg/m^2^; (3) one or more metabolic disorders (e.g., type 2 diabetes, dyslipidemia, hypertension); (4) HbA1c (NGSP) ≤8.5%; and (5) received diet and exercise therapy continuously from at least 4 weeks prior to the start of the intervention; (6) no medications in the period from at least 4 weeks prior to the start of the intervention or taking the same of dose medications continuously from at least 4 weeks prior to the start of the intervention; and (7) after an explanation of the details of the study and understanding the study, the subjects gave their written informed consent for participation. The exclusion criteria are as follows; (1) SGLT2 inhibitor use; (2) severe food allergy; (3) pregnancy, breast-feeding women and expectant women; (4) stage 3–5 diabetic nephropathy; (5) serious hepatic disease excluding fatty liver: an aspartate aminotransferase (AST) or alanine aminotransferase (ALT) level of >2.5 times the upper limit of normal; (6) systolic blood pressure (SBP) ≥150 mmHg or diastolic blood pressure (DBP) ≥100 mmHg; (7) a history of cerebral or cardiovascular events (e.g., cerebral infarction or myocardial infarction) or severe congestive heart failure within 90 days prior to the start of the intervention; (8) proliferative retinopathy; (9) active malignancy; (10) severe mental disorders; and (11) unsuitable for the study.

**Figure 1:**
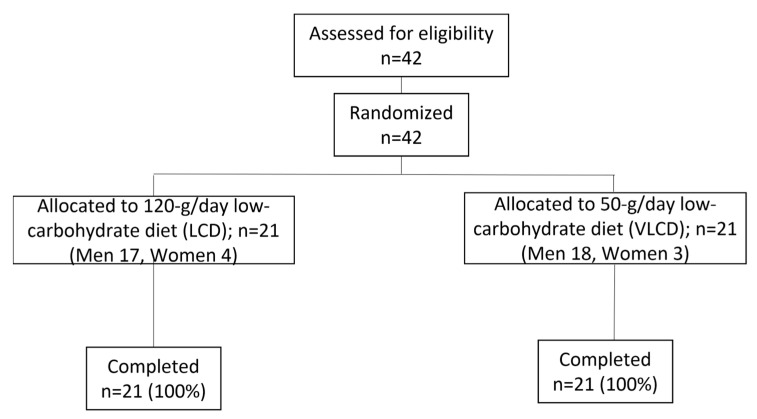
Flow diagram of patient recruitment. Forty-two patients were randomly allocated to either the 120 g/day LCD group or the 50 g/day VLCD group. All patients were followed-up for two months.

The subjects were randomly assigned, using the envelope method, to receive a two-month low-carbohydrate diet intervention (LCD [120 g carbohydrate/day] or VLCD [50 g carbohydrate/day]), as shown in Figure 1. The patients and investigators were not masked to group assignment. 

**Figure 2:**
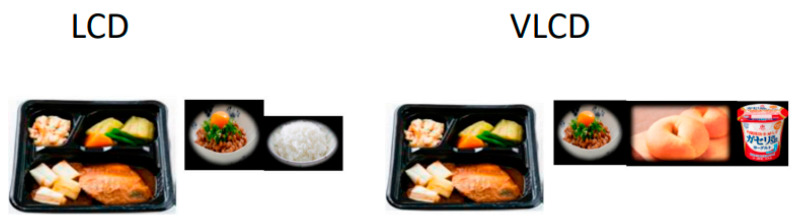
Sample of 1800 kcal test meal.

All study foods (breakfast, lunch, snack and dinner) were provided by the examiners during the study period (Senior Life Create Japan Inc., Tokyo, Japan and RIZAP GROUP Inc., Tokyo, Japan). Meals were carefully designed to provide specified amounts of macronutrients and to contain sufficient fibers; they consisted of seafood and plant-derived protein, such as soybeans and fresh vegetables (Figure 2). All meals were calculated by a dietitian, including energy, carbohydrates, protein, fat (especially the percentage of saturated fatty acids), fiber and salt content. Thereby, the participants’ diet for two months was completely defined. Participants were assigned to report all food to their registered dietitian via an original smart phone app (RIZAP ONLINE^®^) every mealtime so that we could accurately check their compliance. Photos of meals were uploaded to the app after each meal was ingested to confirm that it had been consumed. To avoid any possible influence of the experience and consulting skills of the dieticians in this study, one registered dietician instructed the patients in both groups.

The actual breakfast menu is shown. LCD: Fish and sauté with bean paste, vegetable side dish, 60 g rice and natto with egg. VLCD: Fish and sauté with bean paste, bran bread, natto with egg and yogurt.

The total energy intake was individually calculated based on the ideal body weight and physical activity index. As per the JDS guidelines, total energy intake (kcal) = ideal body weight (kg = height (m) × height (m) × 22) × physical activity index (25–30 with normal intensity activity). The ratio of the three macronutrients in the LCD group was set so that protein accounted for 27%, fat for 47% and carbohydrates for 26% of the total energy. On the other hand, the ratio of the three macronutrients in the VLCD group was set so that protein accounted for 27%, fat for 61%, and carbohydrates for 12% of the total energy (The case of 1800 kcal/day is noted as an example.).

In this study, participants were advised to maintain the same physical activity level as before the study. Each month, body weight and body composition were checked at week −4, week 0 (baseline), week 4 and week 8 using a frequency body impedance analysis (Body Composition Analyzer MC-980A, TANITA, Japan). Regarding physical activity level, an exercise questionnaire was taken before the study began, and the participants pledged not to start any new exercise. Furthermore, at each follow-up (where body composition measurements were taken), the study administrator checked for any changes in exercise practices, such as starting new exercises.

Additionally, blood pressure, HbA1c (NGSP), fasting plasma glucose (FPG), serum ketone bodies (total ketone, 3-hydroxybutyric acid (3OHBA) and acetylacetic acid), kidney function (creatinine (Cr) and estimated glomerular filtration rate (eGFR)), liver function (AST, ALT and γ-glutamyl transpeptidase (γGTP)), lipid profile (total cholesterol (T-Cho), triglycerides (TG), high-density lipoprotein cholesterol (HDL-C) and low-density lipoprotein cholesterol (LDL-C)) and serum C-peptide were checked before and after the study. 

Finally, at the end of the study, we conducted a questionnaire analysis to check the emotional state of the subjects. In the questionnaire, participants were asked three questions: “What was good for you?”, “What was hard?” and “What do you want to do in the future?”. Answers were written freely in Japanese. To quickly and efficiently understand the main content of a collection of documents, we used the JMP pro ver. 15 word cloud software program (SAS Institute Japan, Tokyo, Japan). For the analysis, the text data were pre-processed. Specifically, translation software (Google Translate) was used in 2020. We corrected typographic errors and organized synonyms and compound words. Words that do not make sense on their own (punctuation, particles, etc.) and words used in various contexts, such as “myself,” were excluded. We defined the minimum number of characters per word as two for analysis.

### Statistical Analysis

Comparisons between the two groups were made using Fisher’s direct probability method for categorical variables and Wilcoxon’s rank sum test for parametric variables. *p* values of <0.05 were considered to indicate a statistically significant difference. All analyses were performed using JMP pro ver. 15.

## 3. Results

Of the 42 participants recruited in this study, 21 were assigned to the LCD group (women *n* = 4) and 21 participants (women *n* = 3) were assigned to the VLCD group. All participants in both groups completed the two-month intervention (Figure 1). The baseline characteristics of the participants are summarized in Table 1. There were no statistically significant differences in any of the baseline parameters between the two groups.

### 3.1. The LCD and VLCD Groups Showed an Equal Decrease in BW and Other Body Composition Parameters

**Figure 3:**
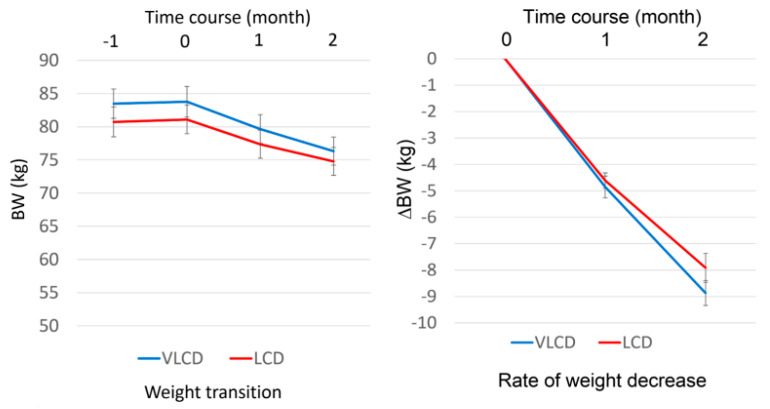
Weight transition and rate of weight decrease after intervention.

At two months after the intervention, the participants showed a significant reduction in BW in comparison to baseline. The BW changes in the two groups are shown in Figure 3; there was no significant difference between the LCD and VLCD groups. The amount of BW change was −6.74 ± 0.42 kg in the LCD group and −7.89 ± 0.51 kg in the VLCD group. In the LCD and VLCD groups, respectively, the change in fat weight was −3.94 ± 0.44 kg and −4.88 ± 0.34 kg, the change in BMI was −2.38 ± 0.19 kg/m^2^ and −2.64 ± 0.15 kg/m^2^ and the change in waist size was −7.79 ± 0.90 cm and −8.67 ± 0.45 cm. There was no significant difference between the two groups in BW change, fat weight, BMI or waist size (Table 2).

Data were expressed as mean ± SD. LCD is indicated with red line. VLCD is indicated with blue line. Left graph shows the weight transition of each group. The vertical axis shows body weight and the horizontal axis shows time lapse. Additionally, −1 month is the result at screening, 0 month is start of the intervention and 1 month and 2 month are one and two months later, respectively. Right graph shows ∆body weight decrease after intervention. 

### 3.2. Lipid Profile

In the LCD group, the change in lipid parameters between before and after the study was as follows: HDL cholesterol, +5 (IQR 0.5 to 8) mg/dL; triglyceride, −103 (IQR −161.5 to −75.5) mg/dL; and LDL-cholesterol, −2 (IQR −17 to 6) mg/dL. In the VLCD group, the change in lipid parameters between before and after the study was as follows: HDL cholesterol, +4 (IQR 1.5 to 9) mg/dL; triglyceride, −98 (IQR −145 to −48) mg/dL; and LDL-cholesterol, 8 (IQR −17.5 to 22.5) mg/dL. There was no significant difference between the two groups in ΔTG, ΔLDL-C, or ΔHDL-C.

### 3.3. Liver Function

After intervention, AST, ALT and γ-GTP were significantly decreased in both groups. In the LCD group, the change in parameters reflecting the liver function was as follows: AST, −6 (IQR −15 to 2) U/L; ALT, −12 (IQR −24.5 to −2) U/L; and γ-GTP, −14 (IQR −40 to −7.5) U/L, in the LCD group (Table 2). In the VLCD group, the change in parameters reflecting the liver function was as follows: AST, −7 (IQR −15 to −3) U/L; ALT, −16 (IQR −35.5 to −5.5) U/L; and γ-GTP, −28 (IQR −52.5 to −13)U/L (Table 2). There was no significant difference between the two groups regarding the ΔAST, ΔALT or γ-GTP.

### 3.4. Glucose Metabolism

During the intervention, HbA1c changed by −0.1% (IQR −0.3% to 0.1%) and −0.2% (IQR −0.4% to 0%) in the LCD and VLCD groups, respectively. Fasting plasma glucose changed by −6 (IQR −22 to 0) mg/dL and −7 (IQR −11 to 1) mg/dL in the LCD and VLCD groups, respectively. The within-group changes in ΔHbA1c and Δfasting plasma glucose were statistically significant. There was no significant difference in these parameters in the between-group comparison (Table 2).

### 3.5. Ketone Bodies

Total ketone bodies, 3-OHBA and acetylacetic acid were significantly increased after the intervention. Comparison of changes between groups showed a greater increase in ketones in the VLCD group than in the LCD group (Table 2).

### 3.6. Other Parameters

Cr did not change in the LCD group; however, Cr was significantly decreased in VLCD group (Table 2). There was a significant change in ΔCr in the between-group comparison.

### 3.7. Questionnaire Survey after the Study

**Figure 4:**
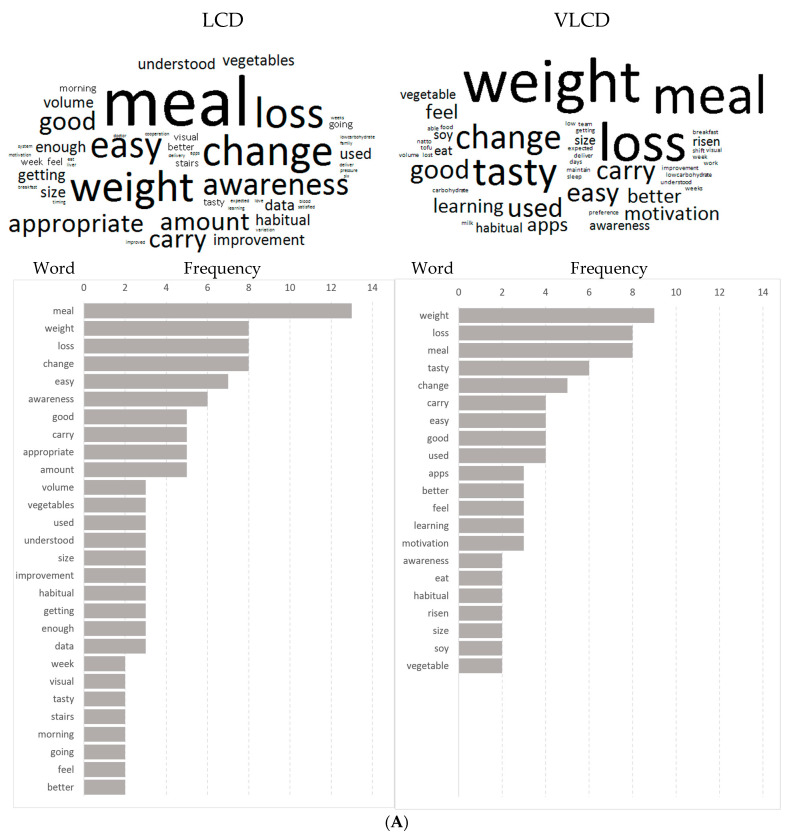

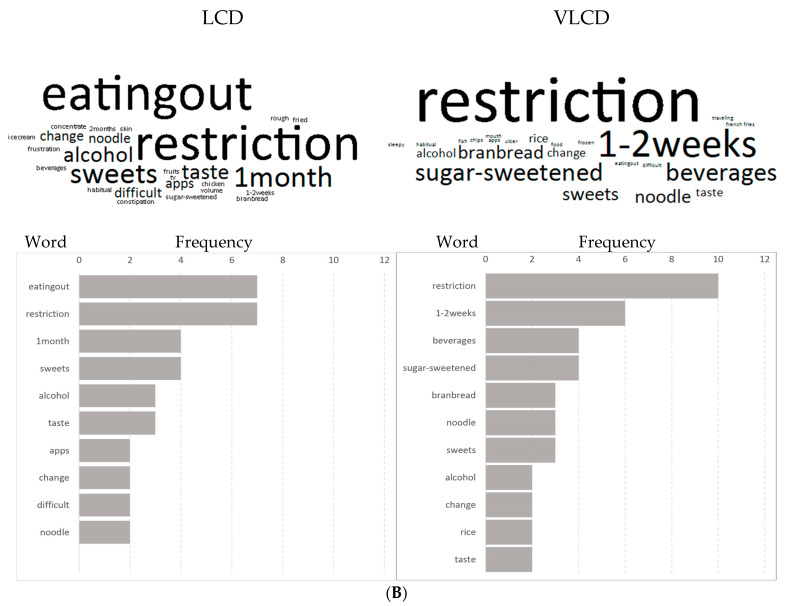

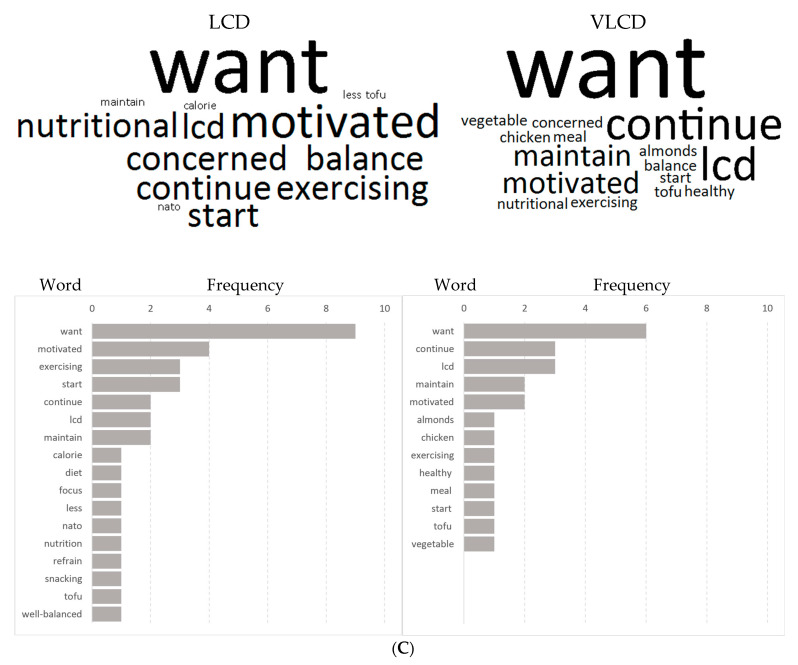
(**A**). The answers to the “What was good for you?*”* were compiled into a word cloud. The larger the letter, the more times the word appeared. The table below shows the number of occurrences of each word found in the word cloud. (**B**) The answers to the “What was hard?” were compiled into a word cloud. The larger the letter, the more times the word appeared. The table below shows the number of occurrences of each word found in the word cloud. (**C**) The answers to the “What do you want to do in the future?” were compiled into a word cloud. The larger the letter, the more times the word appeared. The table below shows the number of occurrences of each word found in the word cloud.

In the LCD group, the following words were seen more than five times for the question, “What was good for you?”: “meal”, “change”, “loss”, “weight”, “easy”, “awareness”, “amount”, “appropriate”, “carry” and “good” (Figure 4A). In contrast, In the VLCD group, the following words were frequently seen: “weight”, “loss”, “meal”, “tasty” and “change”.

Regarding the second question (“What was hard?”), the words “eating out” and “restriction” were the most common words in the LCD group. Other words that were seen more than twice were “1 month”, “sweets”, “alcohol” and “taste”. On the other hand, the following words were frequently seen in the VLCD group: “restriction”, “1–2 weeks”, “beverages”, “sugar-sweetened”, “bran bread”, “noodle” and “sweets” (Figure 4).

Regarding, concerning third question (“What do you want to do in the future?”) “want” was most common in both groups. The words “motivated”, “start” and “exercising” were the next common words in the LCD group. On the other hand, “continue”, “LCD”, “maintain” and “motivated” were the next and third most common words in the VLCD group (Figure 4C). 

## 4. Discussion

We chose two amounts of carbohydrate, 50 g and 120 g, as low-carbohydrate and very-low-carbohydrate diets, respectively. There is no evidence on the suitable amount of carbohydrate in dietary treatment for obese and overweight Japanese patients. A review published in 2015 [5,9,10,11,12,13,14], used two definitions: very-low-carbohydrate diet (20–50 g/day) and low-carbohydrate diet (<130 g/day). However, the amount of dietary intake is difficult to regulate accurately. For example, Sato et al. [7] requested that subjects on a low carbohydrate diet consume 130 g/day carbohydrate, although the median actual carbohydrate intake was 149 g/day (IQR 126–167). In this regard, this study is characterized by its accuracy with regard to ingredients, since all of the test meals were provided to the subjects. Additionally, we adopted a real-time follow-up method using a smartphone app, and a registered dietitian checked carefully; thus, we achieved complete compliance. One clinical study, in which all study meals were supplied to about 10 people per group, showed a high dropout rate for both the high-fat diet and the standard diet. This clinical study was not for weight loss purposes, as the meals were supplied to non-obese subjects [15]. Our study appears to be unique in terms of accuracy and compliance.

Moreover, even if the energy instructions and proportions of macronutrients are the same, different ingredients have different protein properties, which vary widely among fish, meat and vegetables. In the past, many trials [7] have involved subjects voluntarily choosing their own diet and ingredients. As a result, there are likely to be differences among individuals in the nutrients that are given. Foods contain a variety of amino acids and lipids, which are responsible for various metabolic functions in our bodies. Therefore, the specification of energy or basic nutrient composition is not sufficient when comparing the effects of diet on metabolism. With regard to this point, this study defined the conditions of macronutrients and other nutrients. 

There were no significant differences between the LCD and VLCD groups in the change in body composition or change in body weight. Overall, both diets were safe and effective for two months. Moreover, there were no significant differences between the LCD and VLCD groups in the change in the levels of blood parameters during the time that the subjects received the fully-provided diet; however, the change in serum creatinine, eGFR, serum total ketone bodies, 3-OHBA and acetyl acetic acid level was greater in the VLCD group than in the LCD group. Serum creatinine and eGFR might be altered by the different effects of the two diets on the glomerular filtration rate, although we should be cautious in interpreting creatinine levels or eGFR change when there is a significant fluctuation in body weight. Acetyl acetic acid is a typical ketone body, and a very low carbohydrate intake might result in the production of large amounts of ketone bodies. Recently, growing evidence has suggested that elevated ketone bodies have various beneficial effects on health [16,17]. Concerning renoprotective effects, it has been reported that ketone bodies have a direct renoprotective effect, and this was observed in a relatively short period of time of approximately 8 weeks [18]. Although the full extent of ketone body actions remains unclear, several reports show promise, and further research is warranted.

The study meals were also intended to provide a low level of saturated fat, which may promote a more favorable CVD risk profile by elevating HDL-C and reducing TG levels, with comparable reductions in LDL-C, as previously reported [19]. Whether specific nutrients influence this outcome requires further examination.

The questionnaire reflected a high desire to continue the diet in both the VLCD and LCD groups. There were also many statements recognizing weight loss, which may have increased self-esteem due to the achievement of weight loss. In prior studies [20,21], greater weight loss would be associated with a greater improvement in weight-specific health-related quality of life (HRQOL), which consists of a total score and five subscales: physical function, self-esteem, sexual life, work and public distress about one’s weight. The LCD group is more likely than the VLCD group to focus on dietary satisfaction, which may make the diet therapy more likely to be sustainable in the real world. However, the current study provides an example where weight loss results are generally the same if high adherence is maintained, even if the subjects have different impressions of a specific diet when instructed to eat it. The results showed that subjects experienced satisfaction in different domains, either satisfaction with the diet or a sense of accomplishment in the act of weight loss itself. One recent report suggests that high adherence to the Mediterranean diet is accompanied by economic and environmental benefits in addition to high health awareness among the subjects [22]. While dietary satisfaction may be important for high adherence to the diet [23], it is also possible that the resulting outcome of weight loss may foster high awareness of health and improve adherence. In reality, there can be situations where people do not always like the diet or diet regimen they are instructed to follow. In such cases, if weight loss can be achieved through factors other than diet, such as human intervention and guidance not only in person but also through an app, it may result in increased adherence and sustainability.

Various clinical studies have also shown the effectiveness of remote dietary intervention via mobile apps [24,25], which is cost-effective [26]. The app RIZAP ONLINE^®^ we used in this study was originally developed by RIZAP, the company that provided the meals for this trial, and was originally intended for use by Certified Dietitians at gyms to provide dietary advice to their clients. The checking system itself, as well as checking the dietary content, is thought to have contributed to the high level of adherence in this study.

The present study was associated with several limitations. The study population was limited, the study period was relatively short, and the participants could not be blinded due to the characteristics of dietary therapy. However, in this study, high compliance and dietary accuracy were achieved for the first time for obese subjects through meal provision and smartphone follow-up. Intervention provided by high-level professional support and the assistance of the dietary offerings that promoted high compliance was cooperative, and the objective of establishing the characteristics of a low-carbohydrate diet when accompanied by adequate intervention appears to have been achieved.

In conclusion, the present study demonstrated that the intake of an LCD (120 g carbohydrate/day) or VLCD (50 g carbohydrate/day) for two months had similar efficacy and safety in Japanese obese and overweight subjects with metabolic disorders. Significant improvement was achieved in both groups, which suggests that an LCD may have advantageous therapeutic potential for Japanese overweight and obese individuals. Further research is required to establish the longer-term effects. 

## Figures and Tables

**Table 1:** Patients’ characteristics at baseline.

	LCD	VLCD	*p* Value *
*n*	21	21	N/A
Gender (male/female)	17/4	18/3	NS
Age (Years)	44 ± 2	41 ± 2	NS
Height (cm)	166.4 ± 1.7	170.8 ± 1.6	NS
Body weight (kg)	79.2 [75.1–85.1]	83.2 [76.8–91]	NS
Body mass index (kg/m^2^)	29 [27.4–31]	27.6 [26.3–31.5]	NS
Waist (cm)	98 [91.6–103.4]	96.5 [90.8–108]	NS
Body fat (%)	30 [24.2–32.5]	28.1 [25.3–35.6]	NS
SBP (mmHg)	131 [121.5–139]	133 [121–141]	NS
DBP (mmHg)	81 [75–88.5]	77 [70.5–86.5]	NS
AST (GOT) (U/L)	25 [20–40.5]	28 [21–37.5]	NS
ALT (GPT) (U/L)	38 [28–57]	45 [24–72]	NS
γ-GTP (U/L)	46 [26–70]	51 [37–78]	NS
UA (mg/dL)	6.4 [5.3–7.5]	7 [6.5–7.9]	NS
Cr (mg/dL)	0.8 [0.7–0.9]	0.8 [0.7–1]	NS
eGFR (mL/min/1.73 m^2^)	79 [74.4–91.9]	77.1 [70.4–92.9]	NS
TG (mg/dL)	203 [146–259.5]	156 [114–253.5]	NS
T-Cho (mg/dL)	218 [196–251]	214 [190.5–237.5]	NS
HDL-C (mg/dL)	45 [38.5–54]	46 [42.5–54]	NS
LDL-C (mg/dL)	130 [120–157]	135 [118–149.5]	NS
CRP (mg/dL)	0.1 [0.1–0.2]	0.1 [0.1–0.2]	NS
C peptide (ng/mL)	2.5 [1.9–3.3]	2.2 [1.7–3]	NS
FPG (mg/dL)	92 [87–112]	94 [89.5–97.5]	NS
HbA1c (%)	5.6 [5.4–5.8]	5.6 [5.4–5.8]	NS
Total ketone (µmol/L)	50 [37.5, 82]	63 [50, 113.5]	NS
3OHBA (µmol/L)	32 [22.5, 54.5]	40 [28.5, 74]	NS
Acetylacetic acid (µmol/L)	19 [15.5, 19]	26 [18, 39.5]	NS
Antidiabetic drug N (%)	2 (9.5)	1 (4.8)	NS
Antihypertensive agents N (%)	3 (14.3)	2 (9.5)	NS
Lipid-lowering agents N (%)	0 (0)	1 (4.8)	NS
Uric acid treatment N (%)	1(4.8)	1(4.8)	NS
Smoking (Never/Ex/Current) N	7/4/10	3/7/11	NS

Data are expressed as the median (interquartile range). * For between-group comparisons. NS: not significant. N/A: not applicable.

**Table 2:** Data observed at the end of the study period.

	LCD	VLCD	*p* Value *
∆Body weight (kg)	−5.4 [−8.8, −4.7])	−8 [−8.7, −6.1]	NS
∆Body mass index (kg/m^2^)	−2.1 [−3.1, −1.7]	−2.6 [−2.9, −2]	NS
∆Waist (cm)	−6.8 [−8.9, −4.3]	−8.5 [−10.3, −6.5]	NS
∆Body fat (%)	−2.6 [−3.9, −1.5]	−2.8 [−4, −2.4]	NS
∆SBP (mmHg)	−4 [−9.5, −2]	−8 [−17.5, −1.5]	NS
∆DBP (mmHg)	−6 [−13, −1.5]	−5 [–11, –1]	NS
∆AST (U/L)	−6 [–15, –2]	−7 [–15, –3]	NS
∆ALT (U/L)	−12 [−24.5, −2]	−16 [-35.5, −5.5]	NS
∆γ-GTP (U/L)	−14 [−40, −7.5]	−28 [-52.5, −13]	NS
∆UA (mg/dL)	−0.3 [−0.7, 0.8]	−0.5 [−0.9, 0.1]	NS
∆Cr (mg/dL)	−0.01 [−0.04, 0.02]	−0.07 [−0.1, −0.02]	*p* = 0.008
∆eGFR (mL/min/1.73 m^2^)	1 [−1.8, 4.1]	6.9 [1.8, 11]	*p* = 0.01
∆TG (mg/dL)	−103 [−161.5, −75.5]	−98 [−145, −47.5]	NS
∆HDL-C (mg/dL)	5 [0.5, 8]	4 [1.5, 9]	NS
∆LDL-C (mg/dL)	−2 [−17, 6]	8 [−17.5, 22.5]	NS
∆CRP (mg/dL)	−0.02 [−0.06, 0.02]	−0.01 [-0.03, 0.15]	NS
∆C peptide (ng/mL)	−0.6 [−1.6, −0.4]	−0.8 [−1.2, −0.5]	NS
∆FPG (mg/dL)	−6 [−21.5, 0]	−7 [−11, 1]	NS
∆HbA1c (%)	−0.1 [−0.3, 0.1]	−0.2 [−0.4, 0]	NS
∆Total ketone (µmol/L)	140 [67, 485.5]	538 [187.5, 810.5]	*p* = 0.04
∆3OHBA (µmol/L)	98 [49.5, 373.5]	390 [142, 608.5]	*p* = 0.045
∆Acetylacetic acid (µmol/L)	42 [20, 112]	538 [187.5, 810.5]	*p* = 0.03

Data are expressed as the median (interquartile range). Delta represents the data at the end minus that at the baseline. * For between-group comparisons. NS: not significant.

## Data Availability

The datasets generated during and/or analyzed during the current study are available in the OPENICPSR repository (https://www.openicpsr.org/openicpsr/project/186481/version/V1/view, accessed on 4 March 2023).
